# Multi-omics investigation reveals functional specialization of transcriptional cyclin dependent kinases in cancer biology

**DOI:** 10.1038/s41598-022-26860-1

**Published:** 2022-12-28

**Authors:** Micah G. Donovan, Matthew D. Galbraith, Joaquin M. Espinosa

**Affiliations:** 1 Department of Pharmacology, University of Colorado Anschutz Medical Campus, Aurora, CO USA; 2 Linda Crnic Institute for Down Syndrome, University of Colorado Anschutz Medical Campus, Aurora, CO USA

**Keywords:** Transcription, Cancer, Cancer genetics, Oncogenes

## Abstract

Transcriptional addiction is recognized as a valid therapeutic target in cancer, whereby the dependency of cancer cells on oncogenic transcriptional regulators may be pharmacologically exploited. However, a comprehensive understanding of the key factors within the transcriptional machinery that might afford a useful therapeutic window remains elusive. Herein, we present a cross-omics investigation into the functional specialization of the transcriptional cyclin dependent kinases (tCDKs) through analysis of high-content genetic dependency, gene expression, patient survival, and drug response datasets. This analysis revealed specialization among tCDKs in terms of contributions to cancer cell fitness, clinical prognosis, and interaction with oncogenic signaling pathways. CDK7 and CDK9 stand out as the most relevant targets, albeit through distinct mechanisms of oncogenicity and context-dependent contributions to cancer survival and drug sensitivity. Genetic ablation of CDK9, but not CDK7, mimics the effect on cell viability the loss of key components of the transcriptional machinery. Pathway analysis of genetic co-dependency and drug sensitivity data show CDK7 and CDK9 have distinct relationships with major oncogenic signatures, including MYC and E2F targets, oxidative phosphorylation, and the unfolded protein response. Altogether, these results inform the improved design of therapeutic strategies targeting tCDKs in cancer.

## Introduction

Neoplastic transformation is facilitated by genetic and epigenetic changes that alter transcriptional programs, which eventually become essential for cancer cell survival^1^. The recognition of such ‘transcriptional addiction’ has elicited a flurry of research and development activities in pursuit of specific pharmacological agents targeting major transcriptional regulators, such as transcriptional cyclin-dependent kinases (tCDKs)^2–6^. Members of the tCDK family regulate various phases of the RNA polymerase II (RNAPII) transcription cycle, as well as co-transcriptional RNA processing^7,8^. However, it is still unclear how the various members of the tCDK family (i.e., CDK7, - 8, -9, -10, -11A, -11B, -12, -13, -19, -20) are specialized in terms of cellular and organismal functions, both within and outside the transcription cycle. Moreover, it remains to be determined which tCDKs could serve as effective targets to exploit transcriptional addiction in cancer, which cancer types are the most dependent on these tCDKs, and which molecular features confer sensitivity to tCDK inhibition. Therefore, additional investigations are needed to fully reveal the functional specialization of the tCDK family members and to determine their contributions to cancer development.

Several tCDKs have been shown to regulate the RNAPII transcription cycle through their kinase activity, often targeting subunits of the core RNAPII machinery and/or accessory factors. For example, several tCDKs (e.g., CDK7, CDK9) can directly phosphorylate the C-terminal domain (CTD) repeats of the RPB1 subunit of RNAPII, with distinct substrate specificity, thus contributing to control of transcriptional initiation, elongation, and termination^9–11^. Other tCDKs are known to target accessory components of the transcription machinery, such as the Mediator-associated CDK8, which indirectly affects RNAPII transcription through phosphorylation of transcription factors^12^. In addition to specialization, there is both functional cooperation and redundancy among tCDKs. For example, CDK7, acting as part of the TFIIH general transcription factor, has been shown to be required for RNAPII pause-release through T-loop phosphorylation of CDK9, the catalytic subunit of the positive transcriptional elongation factor (P-TEFB), a key regulator of the transition from transcription initiation to elongation^13^. Conversely, other tCDKs may compensate for CDK7 activity^14,15^ and the full network of compensatory interactions among the tCDK family is unclear. Although multiple transcription-related activities have been documented for tCDKs, it is not entirely clear which of these functions sustain transcriptional addiction in cancer cells. Often the same tCDK has been shown to contribute to both oncogenic and tumor suppressive transcriptional programs. For example, CDK8, which works as a co-activator of both the Wnt/B-catenin and the p53 transcriptional programs, among others^12,16–20^. Moreover, some tCDKs may play key roles beyond transcriptional control, as demonstrated for CDK7 in the realm of cell cycle regulation and DNA repair^21–26^. Collectively, these observations reveal many knowledge gaps that hamper the effective design and clinical development of tCDK-based cancer therapies.

Numerous small molecule CDK inhibitors have been reported, including broad-spectrum inhibitors that can target multiple tCDKs at once, as well as selective inhibitors that are claimed to target single kinases or closely related paralogs (e.g., CDK8/19, CDK12/13)^27^. Although broad-spectrum CDK inhibitors showed promising results in preclinical studies and some clinical trials, their clinical application has been limited due to low specificity and significant side effects^4^. In contrast, more selective CDK inhibitors have shown more promise, such as Palbociclib, an inhibitor of the closely related CDK4 and CDK6, which is FDA-approved as a first-line treatment for HR+/HER2- breast cancer^28^. Moreover, tCDK inhibitors such as SY-5609 (CDK7)^29^, THZ1 (CDK7, CDK12/13)^30,31^, THZ2 (CDK7, potentially CDK12/13)^32,33^, AZD4573 (CDK9)^34^, THZ531 (CDK12/13)^35–37^ and cortistatin A (CDK8/19)^17,38^ among several others have demonstrated significant anti-tumor activity in preclinical studies. SY-5609 is currently in clinical trials (ClinicalTrials.gov, NCT04247126) to test its effects in combination with Fulvestrant in advanced hormone receptor-positive, HER2-negative breast tumors. This compound produced strong pro-apoptotic effects in breast and ovarian cancer cell cultures and prevented xenograft tumor formation in vivo. Interestingly, the cytotoxic effects of SY-5609 in cell culture were not observed in non-malignant fibroblast cells and the anti-tumor effects in vivo occurred without concomitant changes in weight loss, indicating a potential therapeutic window^29^. Despite the promising results seen for tCDK inhibitors, it is currently unclear which tCDKs are the most relevant targets across different cancer types and what tumor features represent valid biomarkers of sensitivity to tCDK inhibition. Therefore, there is a critical need for large-scale investigations using models and datasets representative of diverse cancer types.

Within this context, we describe here an investigation of the functional specialization of tCDKs using genetic dependency data from genome-wide knockout screens, global transcriptome analyses, patient survival data, and pharmacological inhibitor response data derived from cell lines and/or tumor samples representing all major cancer types. This analysis revealed differences among tCDKs regarding their effects on cancer cell fitness, tumor progression, and genetic co-dependencies. We found that tCDKs have a wide range of effects on cancer cell viability, with CDK7 and CDK9 being the most essential across different cancer lineages, while other tCDKs display context-dependent essentiality that is independent of cancer type. Analysis of the tCDK gene effect correlations confirmed some expected relationships based on current understanding of the biochemical properties of tCDKs, while also revealing potentially novel functions not necessarily tied to the RNAPII transcription cycle. Notably, these analyses illuminated specialization among close paralogs, i.e., CDK8 versus CDK19, and CDK12 versus CDK13. Finally, analysis of pharmacological inhibitor data and genetic co-dependency data identified gene signatures associated with tCDK dependency that could potentially serve as biomarkers of drug efficacy. Altogether, the analyses and datasets herein provide key insights into roles of tCDKs in cancer biology and resources for follow-up investigations.

## Results

### Human tCDKs have diverse effects on cancer cell viability and tumor growth

**Figure 1:**
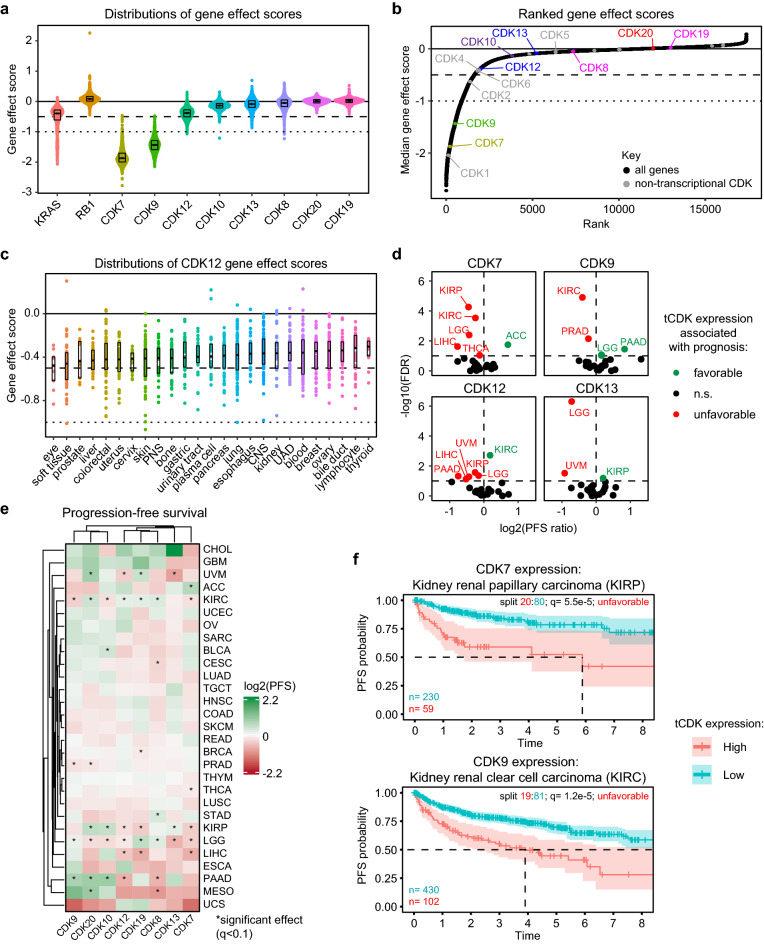
Human tCDKs have diverse effects on cancer cell viability. (**a**) Distributions of gene effect scores for tCDKs, proto-oncogene *KRAS* and tumor suppressor *RB1*. Dashed line indicates threshold for essential gene effect scores (− 0.5). Dotted line indicates strong killing effect (− 1.0). Box plots show median with first and third quartiles at hinges. Whiskers extend to largest/smallest values no further than 1.5*IQR from hinge. (**b**) Ranked plot of median gene effect scores of all genes tested in DepMap data set. The location of all tCDKs (multi-colored) and non-transcriptional CDKs (gray) are denoted. (c) Distributions of gene effect scores for *CDK12* across all lineages with at least 5 representative cell lines. Box plots show median with first and third quartiles at hinges. Whiskers extend to largest/smallest values no further than 1.5*IQR from hinge. (**d**) Volcano plots for prognosis associated with tCDK expression in the TCGA dataset. Results show adjusted (log2) of progression-free survival (PFS) ratio versus adjusted (− log10) false discovery rate (FDR, q value). (**e**) Heatmap showing adjusted (log2) progression-free survival (PFS) associated with tCDKs expression across TCGA cancer lineages. Dendrograms represent results from unsupervised clustering. Asterisks denote significance (FDR < 0.1). (**f**) Results from iterative Kaplan–Meier survival analysis of low (20th percentile) versus high (80th percentile) *CDK7* expression in kidney renal papillary carcinoma (KIRP) and low (19th percentile) versus high (81st percentile) *CDK9* expression in kidney renal clear cell carcinoma (KIRC).

Based on a phylogenetic analysis of full-length CDK protein sequences, tCDKs segregate away from CDKs involved in control of the cell cycle (Supplementary Fig. 1)^39,40^. The tCDKs include CDK7, CDK8, CDK9, CDK10, CDK11A, CDK11B, CDK12, CDK13, CDK19, and CDK20 with three paralog pairs among them (i.e., CDK8/CDK19, CDK11A/CDK11B, and CDK12/CDK13) (Supplementary Fig. 1). To investigate functional specialization within the tCDK sub-family, we analyzed genetic dependency data from the Cancer Dependency Map (DepMap) project^41^, generated via CRISPR-Cas9 knockout screens across 1070 cancer cell lines. In this dataset, negative gene effect scores are indicative of genes required for cell viability and proliferation, as exemplified by the oncogene *KRAS* (Fig. 1a). Genes with effect scores lower than − 0.5 are considered essential for cell viability. On the other hand, positive gene effect scores indicate enhanced growth effects upon knockout of a gene, such as for the tumor suppressor *RB1* (Fig. 1a). Importantly, the widespread essentiality of multiple subunits of the RNAPII enzyme across cancer cells demonstrates their clear dependence on RNAPII-dependent transcriptional processes to sustain viability (Supplementary Fig. 1). The distributions of effect scores for the tCDKs reveal a range of contributions to cancer cell fitness (Fig. 1a, Supplementary Data 1). This is also the case for cell cycle related CDKs (Fig. 1b, Supplementary Data 1) Given that CDK11A and CDK11B could not be knocked out with specific gRNAs, they are not included in this analysis. Notably, knockout of *CDK7* and *CDK9* produces essential gene effect scores (below − 0.5) across all cell lines screened, with even stronger average effects than those observed upon knockout of *KRAS* and the cell cycle-related *CDK4* and *CDK6* (Fig. 1a,b). In fact, the median gene effect scores for *CDK7* and *CDK9* are among the top 3% strongest in the genome, and, among CDKs, the only kinase with stronger contributions to cell viability is CDK1 (Fig. 1b). Whereas *CDK19* and *CDK20* are not essential in any of the cancer cell lines tested, there is a minor proportion of cell lines in which *CDK8* (~ 3%), *CDK10* (~ 1%), *CDK12* (~ 27%) and *CDK13* (~ 3%) are essential. When considering the paralog pairs (i.e., CDK8/19 and CDK12/13), not only is CDK8 essential in 3% of cell lines whereas CDK19 is not essential in any, but also less than half of the cell lines dependent on CDK13 do not require CDK12 (Supplementary Fig. 1). These results indicate functional specialization of the tCDK family members, including within paralog pairs, in the context of cancer cell fitness in the cell culture conditions employed in the DepMap project. To determine if the essentiality of *CDK8/10/12/13* is dependent on cancer type, we examined their gene effect score distributions across 26 different tissues of origin (Fig. 1c, Supplementary Fig. 1). Notably, none of these lineages are distinctly dependent on any of these four kinases. Instead, there are cell lines from several different lineages in which they are essential. This suggests that the conditions dictating essentiality for these four kinases are not lineage-dependent and are likely determined by other molecular and cellular features.

To further investigate the role of tCDKs in the context of human cancer, we analyzed gene expression and survival data from The Cancer Genome Atlas (TCGA) project^42^. In line with the DepMap analysis, higher expression of *CDK7* is associated with worse progression-free survival (PFS) for several cancer types including kidney renal papillary and kidney clear cell carcinoma (KIRP and KIRC, respectively), low-grade gliomas (LGG), liver hepatocellular carcinoma (LIHC), and thyroid carcinoma (THCA) (Fig. 1d–f, Supplementary Data 2). Similarly, *CDK9* expression is associated with unfavorable prognosis in KIRC and prostate adenocarcinoma (PRAD) (Fig. 1d–f). However, for all tCDKs in the analysis, higher expression is associated with either favorable or unfavorable prognosis in at least one cancer type (Fig. 1d,e, Supplementary Fig. 1). For example, high *CDK7* expression is associated with favorable prognosis in adrenocortical carcinoma (ACC) and high *CDK9* expression with favorable prognosis in pancreatic adenocarcinoma (PAAD) and low-grade glioma (LGG) (Fig. 1d,e). Interestingly, expression of *CDK19* and *CDK20*, which did not appear to influence cell survival in the in vitro conditions used to generate the DepMap dataset, are nonetheless associated with better/worse tumor progression free survival (PFS) in multiple tumor types (Fig. 1e, Supplementary Fig. 1). These differences could be potentially explained by cell-autonomous effects of the tCDKs measured in vitro versus cell-extrinsic effects in the context of human tumors and their in vivo microenvironments. This analysis also demonstrated differences between tCDK paralogs regarding their association with the prognosis of different types of cancer, as illustrated by opposite associations between *CDK12* and *CDK13* expression and KIRP prognosis (Fig. 1d,e). Altogether, these results highlight the functional specialization of the tCDKs in terms of cancer cell viability and the potential for context-specific influences on tumor progression.

### Analysis of genetic co-dependencies reveals known and unexpected relationships between tCDKs and cyclins

**Figure 2:**
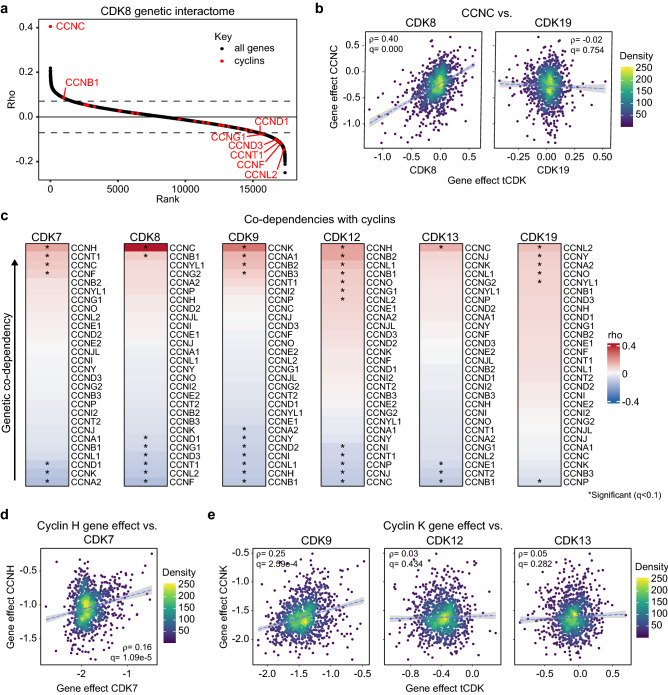
Analysis of genetic co-dependencies reveals predicted versus unexpected relationships between tCDKs and their cyclins. (**a**) Ranked correlations of gene effect scores for *CDK8*. Location of all cyclins depicted in red. Above and below the dashed lines denote significant (FDR < 0.1) positive and negative correlations, respectively. Cyclins with significant (FDR < 0.1) correlations are annotated by name. (**b**) Scatter plot showing gene effects of *CDK8* and *CDK19* versus gene effects of *CCNC* across all 1070 cell lines in the DepMap data set. (**c**) Ranked gene effect correlations between tCDKs and all cyclin genes in the DepMap dataset. Asterisks denote significant (FDR < 0.1) interactions. Arrow shows higher correlation values equate to higher co-dependency scores. (**d**) Scatter plot showing gene effects of *CDK7* versus gene effects of *CCNH* across all 1070 cell lines in the DepMap data set. (**e**) Scatter plot showing gene effects of *CDK9*, *CDK12* and *CDK13* versus gene effects of *CCNK* across all 1070 cell lines in the DepMap data set.

To gain further insight about the functional specialization of tCDKs, we performed an in-depth analysis of their genetic co-dependencies in the DepMap dataset. Co-dependent genes are defined as those whose effects on cell growth are positively or negatively (i.e., inverse co-dependency) correlated, indicating potential biological relationships^43^. First, we mapped the tCDK genetic co-dependencies by ranking Spearman correlations of gene effect scores for all genes in the DepMap dataset against each individual tCDK (Supplementary Data 3). For comparison purposes, we also performed this analysis for all other CDKs (i.e., CDK1-6, 14-17). Given that CDK activation requires binding by a cyclin binding partner, and many biochemical interactions have been defined for tCDKs and various cyclins (Supplementary Fig. 2), we first examined the association of all cyclins previously reported to bind to and regulate CDK activity (Fig. 2a, Supplementary Fig. 2). Notably, in some instances the known cyclin partner is the top positive co-dependency overall, such as in the case for association of cyclin C (*CCNC*) with *CDK8* (Fig. 2a,b). In other instances, the known cyclin partner is the top co-dependency among the cyclins, such as in the case of association of cyclin H (*CCNH*) with *CDK7* and cyclin K (*CCNK*) with CDK9 (Fig. 2c–e). These results demonstrate the capacity of genetic co-dependencies to highlight *bona fide* functional relationships between genes. However, for other tCDKs, the known cyclin partner shows no significant co-dependency, such as the case for *CCNC* versus *CDK19* or *CCNK* versus *CDK12* and *CDK13* (Fig. 2b,c,e, Supplementary Fig. 2). Thus, whereas depletion of a tCDK or its partner cyclin may have largely similar effects across hundreds of cell lines in some instances (e.g., *CDK8* and *CCNC*), this is not always the case (e.g., *CDK19* and *CCNC*) (Fig. 2b,c). Some tCDKs had stronger co-dependencies with cyclins other than their established binding partners. For example, in the cases of *CDK12* and *CDK13*, *CCNH* and *CCNC* are the top correlated cyclins, respectively (Fig. 2c,e, Supplementary Fig. 2). Interestingly, for CDK12, cyclins H, B2, L1, B1, O, G1 and L2 all rank higher than cyclin K. Similarly, for *CDK19*, multiple cyclins rank above *CCNC*, including significant co-dependencies with cyclin L2 (*CCNL2*), Y *(CCNY*), A2 (*CCNA2*), O (*CCNO*) and YL1 (*CCNYL1*) (Fig. 2c, Supplementary Fig. 2). Other unexpected observations include significant co-dependency between *CCNL2* and *CDK10*, and *CCNL2*, cyclin B2 (*CCNB2*), and *CCNK* with *CDK20* (Supplementary Fig. 2). Altogether, these results demonstrate the capacity for the DepMap dataset to both highlight known functional interactions of the tCDKs and also identify potentially novel biological relationships including possible alternative CDK/cyclin partnerships.

### tCDKs exhibit differential genetic co-dependencies with the RNAPII machinery

**Figure 3:**
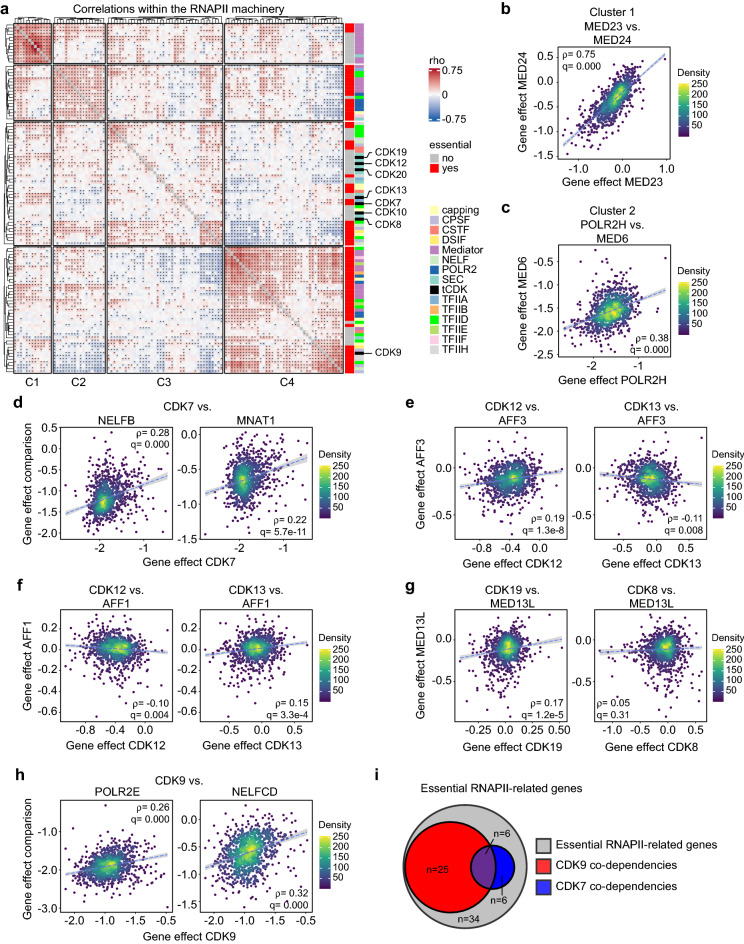
tCDKs exhibit differential genetic co-dependencies with the RNAPII machinery. (**a**) Heatmap depicting gene effect correlations between tCDKs and various genes involved in RNAPII-dependent transcription. Asterisks denote significant (FDR < 0.1). Essential genes (red, right-side annotation) are determined by median gene effect scores of < − 0.5 across all cell lines. In the multi color-coded annotation to the right of the heatmap, colors indicate diverse protein complexes. Dendrograms represent results from unsupervised clustering analysis. (**b**) Scatter plot comparing gene effect scores of *MED23* and *MED24* across all 1070 cell lines in the DepMap data set. (**c**) Scatter plot comparing gene effect scores of *POLR2H* and *MED6* across all 1070 cell lines in the DepMap data set. (**d**) Scatter plot comparing gene effect scores of *CDK7* to *NELFB* and *MNAT1* across all 1070 cell lines in the DepMap data set. (**e**) Scatter plot comparing gene effect scores of *CDK12* and *CDK13* to *AFF3* across all 1070 cell lines in the DepMap data set. (**f**) Scatter plot comparing gene effect scores of *CDK12* and *CDK13* to *AFF1* across all 1070 cell lines in the DepMap data set. (**g**) Scatter plot comparing gene effect scores of *CDK8* and *CDK19* to *MED13L* across all 1070 cell lines in the DepMap data set. (**h**) Scatter plot comparing gene effect scores of *CDK9* to *POLR2E* and *NELFCD* across all 1070 cell lines in the DepMap data set. (**i**) Venn diagram comparing significant positive co-dependencies (rho > 0, FDR < 0.1) of CDK7 and CDK9 among essential (median gene effect < − 0.5) transcription-related genes shown in (**a**).

Next, we turned to the RNAPII transcription cycle. This process is regulated by diverse events at several steps involving the kinase activity of multiple tCDKs, whereby RNAPII and several transcriptional co-factors are direct phosphorylation targets^7^. Thus, we assessed co-dependencies between tCDKs and genes encoding key factors involved in the transcription cycle, including RNAPII subunits, general transcription factors (GTFs, i.e., TFIIA, B, D, E, F and H), the Mediator complex, regulators of RNAPII pause, release, and elongation (negative elongation factor, NELF; DRB Sensitivity Inducing Factor, DSIF; and the super elongation complex, SEC), as well as the capping and 3’ processing machineries (cleavage and polyadenylation specificity factor, CPSF; and cleavage stimulation factor, CSTF) (Fig. 3a, Supplementary Fig. 3, Supplementary Data 4). Analyzing the full matrix of correlations by unsupervised clustering shows four distinct gene clusters (Fig. 3a). Cluster 1 does not contain any tCDKs and is comprised of mostly non-essential subunits of the Mediator complex. This cluster has the strongest associations in this analysis, as illustrated by the co-dependencies between *MED23* and *MED16* with *MED24*, components of the Mediator tail module (Fig. 3b, Supplementary Fig. 3). Previous studies have also indicated tail subunits are not essential for cell viability, despite the tail being the main docking site for DNA-binding transcription factors^44^. Interestingly, in yeast, the Mediator tail module is not required for recruitment to DNA promoters and only a small subset of genes is affected by tail depletion^45,46^. Cluster 2, which like Cluster 1 does not contain any tCDKs, consists of essential subunits mainly from Mediator and RNAPII, as illustrated by co-dependency between *MED6* and *POLR2H* (Fig. 3c) and *MED14* and *MED6* (Supplementary Fig. 3). This cluster also contains a few essential subunits from TFIIA, TFIID, TFIIF, the capping complex, and the SEC (Fig. 3a). Interestingly, except for CDK9, all the tCDKs group within Cluster 3, which does not contain any RNAPII subunits and is largely dominated by non-essential accessory factors. Various associations within this cluster provided confidence in our analysis, including co-dependency between *CDK7* and *NELFB* and various components of TFIIH including *MNAT1* (Fig. 3d). Along with cyclin H, CDK7 and MNAT1 form the heterotrimeric CDK activating kinase (CAK) complex, which activates the cell cycle-related CDKs 1, 2, 4, and 6 through T-loop phosphorylation^25,47^. The CAK complex also associates with TFIIH wherein the kinase activity of CDK7 regulates various transcription-related processes including CTD phosphorylation of RNAPII and the recruitment of NELF to transcription start sites^48^. This analysis also revealed surprising differences between closely related tCDK paralog pairs. For example, within Cluster 3, the strongest interaction for *CDK12* is *AFF3*, which has inverse co-dependency with *CDK13* (Fig. 3e). The opposite relationship is observed with *AFF1*, which is the strongest co-dependency for *CDK13* in this cluster and displays an inverse co-dependency with *CDK12* (Fig. 3f). *AFF1* and *AFF3* encode evolutionarily divergent members of the AF4/FMR2 family of transcription elongation factors, which have been shown to regulate distinct target genes and have differential expression across tissues and throughout development^49^. Taken together this could suggest context-dependent participation of CDK12/13 in transcription regulation by SECs. *CDK8* and *CDK19* also showed surprising differences regarding their interactions with members of the Mediator complex (Fig. 3a, Supplementary Fig. 3). Whereas *CDK19* shows co-dependency with several *MED* genes, as illustrated by the interaction with *MED13L*, a component of the Mediator kinase module and itself a paralog of MED13, this is not the case for *CDK8* (Fig. 3g). This may indicate prominent roles for CDK8 (and by association cyclin C) in other cellular processes, beyond its well-documented positive and negative regulatory effects on transcription as part of the Mediator complex. This could also indicate Mediator-independent roles for CDK8 in transcription regulation.

Finally, *CDK9* falls within Cluster 4, which contains several RNAPII subunits and essential genes from other key transcriptional co-factors (Fig. 3a). Consistently, *CDK9* shows the strongest genetic co-dependencies among the tCDKs with essential subunits from most of these transcriptional complexes as illustrated by *POLR2E* (RNAPII), *NELFCD* (NELF), *TAF2* (TFIID) and *NCBP1* (capping), among other examples (Fig. 3h, Supplementary Fig. 3). In line with these observations, CDK9 has been shown to play a pivotal role in controlling transcription elongation, in part, through regulating the interaction between the NELF complex and the transcription machinery^50^. NCBP1 has also previously been identified as a substrate for CDK9 kinase activity^51^. Interestingly, whereas *CDK9* is co-dependent with several subunits of RNAPII, this is not the case for *CDK7*, which shows inverse co-dependency with multiple subunits (Supplementary Fig. 3). Moreover, of the 71 genes considered essential among this transcription-related gene set, ~ 44% show positive co-dependency with *CDK9*, whereas < 20% are co-dependent with *CDK7* (Fig. 3i).

Collectively, these observations suggest that while cancer cells respond similarly, in terms of cell viability in vitro, to the loss of CDK9 and key factors controlling transcription, this is not the case for the majority of tCDKs, including CDK7. This could imply that the dependence of cancer cells on CDK7 may not be primarily due to its transcription-related activities.

### Pathway analysis of genetic interactomes predicts novel functions for tCDKs

**Figure 4:**
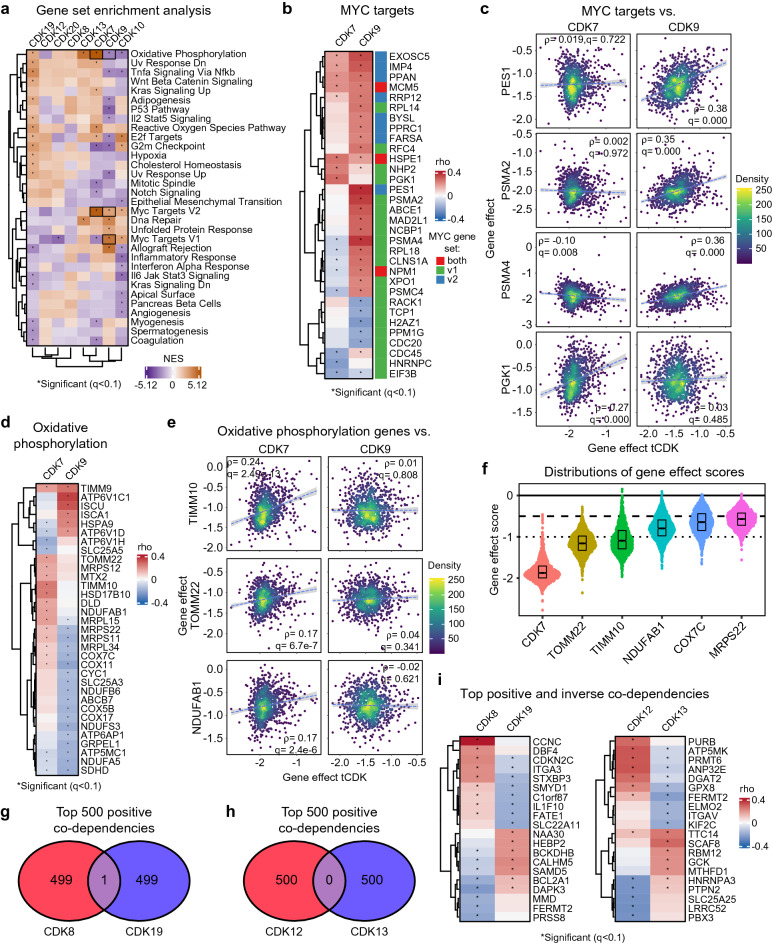
Pathway analysis of genetic interactomes predicts novel functions for tCDKs. (**a**) Heatmap showing normalized enrichment scores (NES) from Hallmark gene set enrichment analysis of ranked tCDK gene effect correlations. Asterisks denote significant (FDR < 0.1). Dendrograms represent results from unsupervised clustering analysis. (**b**) Heatmap showing gene effect correlations for *CDK7* and *CDK9* versus representative genes from the Hallmark MYC Target V1 and V2 datasets. Asterisks denote significance. In the color-coded annotation to the right of the heatmap, green denotes genes from the V1 dataset, blue denotes the V2 dataset, red indicates genes from both V1 and V2. (**c**) Scatter plots comparing gene effect scores of *CDK7* and *CDK9* to select genes from the MYC targets datasets. (**d**) Heatmap showing gene effect correlations for *CDK7* and *CDK9* vs. representative genes from the Hallmark Oxidative Phosphorylation data set. Asterisks denote significance (FDR < 0.1). Dendrograms represent results from unsupervised clustering analyses. (**e**) Scatter plots comparing gene effects of *CDK7* and *CDK9* to select genes involved in oxidative phosphorylation. (**f**) Distributions of gene effect scores for select co-dependencies of *CDK7* involved in oxidative phosphorylation. Dashed line indicates threshold for essential gene effect scores (− 0.5). Dotted line indicates strong killing effect (− 1.0). Box plots show median with first and third quartiles at hinges. Whiskers extend to largest/smallest values no further than 1.5*IQR from hinge. (**g**,**h**) Comparison of top 500 positive gene effect correlations between the tCDK paralog pairs *CDK8/19* (**g**) and *CDK12/13* (**h**). (**i**) Heatmaps comparing gene effect correlations of *CDK8* to *CDK19* and *CDK12* to *CDK13* with their top 5 positive and inverse co-dependencies. Asterisks denote significance (FDR < 0.1). Dendrograms represent results from unsupervised clustering analyses.

We next extended our investigation of the tCDK genetic interactome outside of the RNAPII transcription cycle by completing gene set enrichment analysis (GSEA) of hallmark pathways against the ranked genome-wide co-dependencies of each tCDK (Fig. 4a, Supplementary Data 5). Overall, each tCDK presents a unique pattern of pathway enrichment in its genetic interactome, with multiple examples of signaling pathways displaying significant genetic interactions with a single tCDK. Given the fact that CDK7 and CDK9 are more essential for cancer cell fitness relative to the other tCDKs, we focused on gene signatures enriched among their positive co-dependencies with potential oncogenic roles. Both *CDK7* and *CDK9* showed co-dependencies with MYC target genes, albeit with important differences at the gene level (Fig. 4b,c). Expectedly, many MYC target genes are essential for cancer cell fitness (Supplementary Fig. 4). Whereas both kinases show co-dependencies with the MYC V2 gene set, only *CDK9* shows co-dependencies with the MYC V1 set (Fig. 4a–c, Supplementary Fig. 4). In fact, *CDK7* co-dependencies are negatively enriched for MYC V1 targets (Fig. 4a). Moreover, at the gene level, their co-dependencies with these oncogenic gene signatures are clearly different (Fig. 4b). For example, *CDK9*, but not *CDK7*, shows co-dependency with *PES1* (Pescadillo homolog 1), a key pro-proliferation factor involved in pre-processing of the 60 s ribosome subunit. Additionally, *CDK9*, but not *CDK7*, shows co-dependencies with subunits of the proteasome, such as *PSMA2* and *PSMA4* (Fig. 4b,c). In contrast, *CDK7* but not *CDK9*, shows co-dependency with *PGK1*, a key MYC target gene involved in metabolic reprogramming (Fig. 4b,c). This could indicate that CDK7 and CDK9 contribute to cancer cell fitness through regulation of different aspects of the MYC transcriptional network. Alternatively, it could imply different cancer cell lines maintain expression of MYC targets through differing means, which imparts distinct dependencies. Beyond MYC signatures, other important differences include significant co-dependencies between *CDK9*, but not *CDK7*, and several essential genes involved in the unfolded protein response (UPR) (Fig. 4a, Supplementary Fig. 4). Conversely, *CDK7*, but not *CDK9*, shows co-dependencies with multiple essential factors in the Oxidative Phosphorylation gene signature, including subunits of the electron transport chain complexes (e.g., *COX7C*, *COX11*, *NDUFAB1*) and several essential genes encoding mitochondrial proteins (e.g., *TIMM10*, *TOMM22*, and *MRPS22*) (Fig. 4d–f). Therefore, whereas both CDK7 and CDK9 may contribute to cancer cell fitness through differential interactions with the MYC network, they display clearly specialized relationships with other signaling pathways relevant for cancer development.

Beyond *CDK7* and *CDK9*, this pathway analysis also identified clear distinctions between the tCDK paralog pairs *CDK8/19* and *CDK12/13* (Fig. 4a). For example, whereas the co-dependencies for *CDK19* are positively enriched for several hallmark pathways, this is not the case for *CDK8* (Fig. 4a). Among their top 500 co-dependencies, *CDK8* and *CDK19* have a single common interaction (Fig. 4g), the armadillo repeat domain containing protein 2 (*ARMC2*), which has been found to be essential for sperm-tail biogenesis in both humans and mice^52^. Conversely, *CDK12* and *CDK13* have no common co-dependencies among their top 500 interactions (Fig. 4h). Analysis of the 5 strongest positive and inverse co-dependencies for each of the paralog pairs demonstrated that the relationship is often opposite for the same gene (e.g., *CDKN2C* vs. *CDK8/19*, *ATP5MK* vs. *CDK12/13*) (Fig. 4i). Importantly, previous biochemical-based studies have documented opposite roles for CDK8 and CDK19 on transcriptional regulation in certain contexts^53^.

Collectively, these results suggest that tCDKs have distinct and specialized genetic interactions with diverse signaling pathways, beyond the RNAPII transcription cycle, including within closely related kinases, which could potentially explain their differential contributions to cancer cell viability and cancer development.

### Identification of gene signatures associated with sensitivity to pharmacological tCDK inhibition

**Figure 5:**
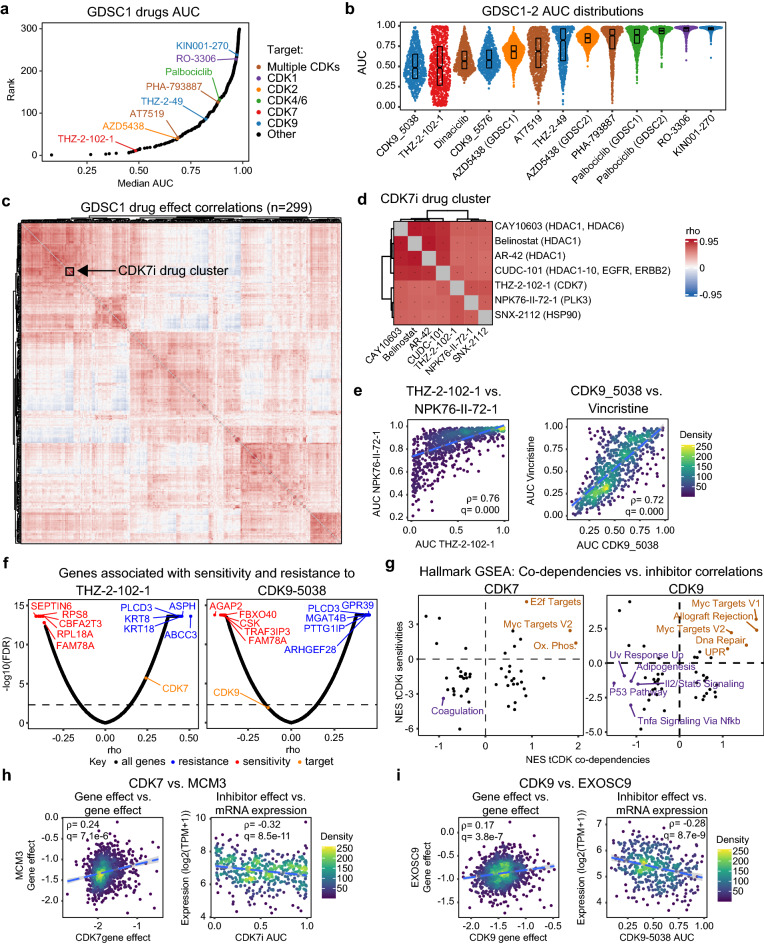
Identification of gene signatures associated with sensitivity to pharmacological tCDK inhibition. (**a**) Ranked median area under the curve (AUC) values for all drugs in the GDSC1 data set. Drugs targeting CDKs are color-coded and annotated. (**b**) Distributions of AUC values for drugs targeting CDKs across all cell lines in the GDSC1 and GDSC2 data sets. Box plots show median with first and third quartiles at hinges. Whiskers extend to largest/smallest values no further than 1.5*IQR from hinge. (**c**) Heatmap showing full matrix of correlations of AUC between all drugs in the GDSC1 dataset. Dendrograms represent results from unsupervised clustering analyses. Cluster containing the CDK7 inhibitor THZ-2-102-1 is blocked off in a black square. (**d**) Magnification of a cluster from (c) containing the CDK7 inhibitor THZ-2-101. Asterisks denote significance (FDR < 0.1). Dendrograms represent results from unsupervised clustering analyses. (**e**) Scatter plots comparing AUC of CDK7 inhibitor THZ-2-102 to PLK3 inhibitor NPK76-II-72-1 (left) and CDK9 inhibitor CDK9_5038 to microtubule destabilizer vincristine (right). (**f**) Volcano plots depicting correlations between CDK7 (THZ-2-102-1) and CDK9 (CDK9-5038) inhibitors versus genome-wide mRNA expression. The top 5 sensitivity (rho < 0, FDR < 0.1) and resistance (rho > 0, FDR < 0.1) genes are shown in red and blue, respectively. Drug targets are highlighted in orange. (**g**) Scatter plots comparing normalized enrichment scores (NES) from Hallmark gene set enrichment analysis of ranked tCDK gene effect correlations (x-axis) versus ranked correlations of mRNA expression versus tCDK inhibitor AUC (y-axis). (**h**) Scatter plots comparing gene effect of CDK7 to that of MCM3 (left) and drug effect of CDK7 inhibitor (THZ-2-102-1) versus expression of MCM3 (right). (**i**) Scatter plots comparing gene effect of CDK9 to that of EXOSC9 (left) and drug effect of CDK9 inhibitor (CDK9-5038) versus expression of EXOSC9 (right).

To gain a pharmacological perspective into mechanisms of specialization among tCDKs, we investigated the Genomics of Drug Sensitivity in Cancer (GDSC) datasets, which contain data on the sensitivity of hundreds of cancer cell lines (GDSC1, n = 985; GDSC2, n = 809) to hundreds of drugs (GDSC1, n = 299; GDSC2, n = 173). In these datasets, the only tCDKs targeted by specific inhibitors are CDK7 (targeted by THZ-1-102-1 in GDSC1) and CDK9 (targeted by THZ-2-49, and KIN001-270 in GDSC1, and CDK9_5576 and CDK9_5038 in GDSC2). However, the datasets also contain information about inhibitors of CDK1 (RO-3306), CDK2 (AD5438), CDK4/6 (Palbociclib), as well as broad-spectrum CDK inhibitors (e.g., PHA-793887, AT7519). Remarkably, when all drugs in the GDSC1 dataset are ranked by their effects on cell viability (i.e., area under the curve, AUC), the CDK7 inhibitor THZ-2-102-1 has the 11th strongest pharmacological effect with a median AUC of 0.49 (Fig. 5a). On the other hand, the four CDK9 inhibitors have a wide range of effects on cancer cell growth. Whereas the CDK9 inhibitor CDK9_5038 is ranked 4th among all drugs in the GDSC2 dataset (Supplementary Fig. 5), the compound KIN001-270 ranks 238th in GDSC1 (Fig. 5a). In line with our analysis of genetic dependency data, the CDK7 inhibitor THZ-2-102-1 and the CDK9 inhibitor CDK9_5038 have stronger effects than compounds targeting major CDKs involved in cell cycle progression such as CDK2 (e.g., AZD5438) and CDK4/6 (e.g., Palbociclib), and those targeting a broad spectrum of CDKs (Fig. 5a, Supplementary Fig. 5). Nevertheless, there is a wide distribution of effects for all these CDK inhibitors across cancer cell lines of diverse origins (Fig. 5b), which enabled us to complete correlation analyses both between drug effects and versus matching gene expression data generated from these cell lines (Supplementary Data 6 and 7).

First, we investigated the relationship between CDK inhibitors and all other drugs in the datasets by defining Spearman correlations and visualizing similarities via unsupervised clustering (Fig. 5c, Supplementary Fig. 5). Notably, the CDK7 inhibitor THZ-2-102-1 falls within a drug cluster that includes a PLK3 inhibitor (NPK-II-72-1), an HSP90 inhibitor (SNX-212), and several HDAC inhibitors (e.g., Belinostat, CAY-10603) including CUDC-101, which is reported to also target human epidermal growth factor receptors 1 and 2 (EGFR1 and HER2, respectively) (Fig. 5c–e). Importantly, the action of all these targets can be tied to cell cycle processes. For example, PLK3 is required for S-phase entry^54^, HSP90 chaperones a myriad of proteins involved in cell cycle progression (including PLKs)^55^, HDACs repress key cell cycle regulators such as p53 and p21^56^, and EGFR signaling activates various downstream pathways that stimulate cell cycle progression including RAS-RAF-MEK-ERK-MAPK and AKT-PI3K-mTOR^57^. In contrast, the CDK9 inhibitors CDK9_5038 and CDK9_5576 associate with each other and within a cluster that includes a CDK2 inhibitor (AZD5438) and a broad spectrum CDK inhibitor (Dinaciclib, reported to target CDK1, CDK2, CDK5, CDK9) (Supplementary Fig. 5). This cluster is also comprised of various microtubule destabilizers (e.g., Vincristine, Vinblastine, Vinorelbine) in addition to an inhibitor of RNA helicase A (YK-4-279), which has also been reported to act as a microtubule destabiliser^58^, and kinesin proteins (Eg5_9814), which are important regulators of microtubule dynamics and cell division processes^59^ (Fig. 5d,e, Supplementary Fig. 5). These results suggest that CDK7 inhibitors elicit a response in cancer cells similar to compounds that target regulators of cell cycle progression (PLK3, HSP90, HDACs, EGFR), whereas CDK9 inhibitors produce responses similar to drugs targeting mitosis (i.e., microtubule stability and dynamics).

Next, we investigated gene signatures associated with sensitivity or resistance to the various CDK inhibitors in the GDSC datasets. To this end, we performed pairwise Spearman correlation analyses of CDK inhibitor AUC versus RNA expression data for all available genes in matched cell lines (Fig. 5f, Supplementary Fig. 5, Supplementary Data 7). Given that lower AUC is associated with an anti-proliferative effect, genes with negative correlations to CDK inhibitor AUC were considered sensitivity genes whereas genes with positive correlations were considered resistance genes. We focused this analysis on inhibitors of CDK7 (THZ-2-102-1), CDK9 (CDK9_5038), CDK1 (RO-3306), CDK2 (AZD5438) and CDK4-6 (Palbociclib). Although data for AZD5438 and Palbociclib were available in both GDSC1 and GDSC2 datasets, we used those from GDSC2 as they represented the most up-to-date data for these drugs. Notably, this analysis demonstrated that the top resistance gene for THZ-2-102 is *ABCC3*, a well-known multi-drug resistance gene^60^ (Fig. 5f). A relatively uncharacterized gene, *FAM78A*, was among the top sensitivity genes for both the CDK7 inhibitor THZ-2-102-1 and the CDK9 inhibitor CDK9_5038 (Fig. 5f). Additionally, both inhibitors of CDK7 (THZ-2-102-1) and CDK4/6 (Palbociclib) display ribosomal subunits among their top sensitivity genes (Fig. 5f, Supplementary Fig. 5). To gain a more global perspective of the signaling pathways associated with sensitivity or resistance to each compound, we performed GSEA on the AUC versus inhibitor correlations for each CDK inhibitor (Supplementary Fig. 5). For comparison purposes, we also included the most similar drug (by Spearman correlation) to each of these CDK inhibitors (i.e., NPK76-II-72-1 for THZ-2-102-1, Vincristine for CDK9-5038, JNK-Inhibitor-VII for RO-3306, Oxaliplatin for Palbociclib, and VSP34_8731 for AZD5438). GSEA was carried out on the inverse of the correlations so that positive and negative enrichment represented sensitivity and resistance genes, respectively. Unsupervised clustering of Hallmark GSEA results showed the CDK7, CDK9 and CDK1 inhibitors cluster closest with their most related drugs whereas the CDK4/6 and CDK2 inhibitor cluster closest with each other (Supplementary Fig. 5). However, overall, there is significant overlap between the CDK7, CDK9, CDK4/6 and CDK2 inhibitors regarding the most positively and negatively enriched pathways and clear non-overlap with the CDK1 inhibitor. Interestingly, whereas there is marked non-overlap between CDK7 and CDK9 in terms of pathways positively/negatively enriched among their genetic co-dependencies (Fig. 4a), the overall pattern of signaling pathways associated with their pharmacological inhibition is more similar. This could be due to the fact that pharmacologic suppression only inhibits tCDK kinase activity, whereas genetic ablation also removes accessory functions of the tCDKs, such as scaffolding. Nevertheless, several of the gene signature pathways that were positively enriched among the genetic co-dependencies of *CDK7* and *CDK9* are also enriched among the genes associated with sensitivity to their pharmacologic inhibitors (Fig. 5g). For CDK7, these common signatures include E2F targets, MYC targets (V2) and genes involved in oxidative phosphorylation, whereas the common signatures for CDK9 include MYC targets (V1 and V2), DNA repair, the UPR and allograft rejection (Fig. 5g). Thus, these shared gene signatures could be considered high-confidence modulators of the cellular response to loss of tCDK activity. An example of a gene whose expression is associated with both *CDK7* genetic dependency and sensitivity to CDK7 pharmacological inhibition is *MCM3*, a subunit of the hexameric MCM (mini-chromosome maintenance) complex critical for initiation of DNA replication and a known E2F target gene (Fig. 5h). The analogous example for CDK9 is *EXOSC9*, a component of the RNA exosome complex involved in the UPR, which displays genetic co-dependency with *CDK9* and whose expression is associated with sensitivity to CDK9 inhibition.

Altogether, these results highlight the existence of signaling pathways differentially associated with the activity of different tCDKs, with clear potential to illuminate both mechanisms of tCDK action as well as development of more effective tCDK-based cancer therapies.

## Discussion

As a result of evolutionary divergence and specialization, mammalian cells evolved two functionally distinct classes of CDK, those that are involved mostly in control of the cell cycle (e.g., CDK1, -2, -4, -5, -6) and tCDKs^39^. Despite the recognition that transcriptional addiction is a valid target for cancer therapy, the cell cycle related CDKs, most prominently CDK4/6, have received more attention in terms of development as therapeutic targets. Recent efforts have led to identification of specific inhibitors of select tCDKs (CDK7, CDK9) as well as closely related paralog pairs (CDK8/19, CDK12/13), which are in various stages of clinical development^4^. Nonetheless, several factors continue to limit the therapeutic targeting of tCDKs, including an incomplete understanding of their requirement for tumor development and maintenance across diverse cancer types, as well as the lack of helpful biomarkers of drug efficacy. To help address these knowledge gaps, we performed a cross-omic analysis of the functional specialization of the tCDK family in cancer cells.

In this study we used genetic dependency data, TCGA survival analysis, transcriptomics, and pharmacological inhibitor sensitivity data to assess the specialization of the tCDK family members in cancer. We used correlations of genetic dependency data (co-dependencies) to investigate relationships of the tCDK family members and ascribe mechanisms owing to their effects on cancer cell viability. Our analysis revealed CDK7 and CDK9 to be the most oncogenic tCDKs, potentially more so than CDK4 and CDK6. Whereas previous studies documented negative effects on cancer cell viability with pharmacologic^32,34^ and genetic^31,61^ suppression of both CDK7 and CDK9 in specific cancer models, our analysis of DepMap data indicates both kinases are essential across all 1070 cell lines tested, with stronger average effects than both CDK4 and CDK6. The higher potency associated with CDK7/CDK9 inhibition was also apparent in our analysis of the drug sensitivity datasets. Our analysis of their genetic co-dependencies suggested the oncogenicity of CDK7 and CDK9 may be due to potentially distinct mechanisms. For example, whereas loss of CDK9 produces responses in cancer cells that resemble the loss of key components of the transcriptional machinery (e.g., RNAPII subunits), this is not the case for CDK7. This suggests that the oncogenicity of CDK7 may not be primarily linked to sustaining transcriptional addiction. In support of this notion, previous studies have shown CDK7 to be dispensable for RNAPII CTD phosphorylation and global mRNA transcription^62–64^. Deletion of *Cdk7* in mouse embryonic fibroblasts triggers cell cycle arrest, independent of changes in global RNAPII CTD phosphorylation levels^62^. Moreover, selective inhibition of CDK7 has been shown to induce DNA replication stress and genomic instability causing cell cycle arrest without affecting global RNAPII phosphorylation in tumor cells^63^. CDK7 has a unique role among the tCDK family in that it is known to affect both transcriptional and cell cycle related processes as part of the CAK complex^21–25^. Downstream of CDK4/6 activation by the CAK complex, Rb-dependent repression of E2F is relieved, which triggers the expression of various genes promoting cell cycle progression. Although loss of CDK7 does not resemble the loss of the transcriptional machinery, our co-dependency analysis revealed resemblance to loss of E2F targets and MYC targets. Moreover, both signatures are enriched among genes associated with sensitivity to CDK7 inhibitors. Interestingly, previous studies showed suppression of CDK7 specifically hampers the expression of E2F^64^ and MYC-driven^26^ transcriptional programs, imparting anti-tumorigenic effects. Our clustering analysis of drug effect correlations showed that the effect of THZ-2-102-1, a selective CDK7 inhibitor, had similar efficacy to drugs targeting key regulators of cell cycle progression. Taken together, these results could suggest the oncogenicity of CDK7 is more likely ascribed to its role in cell cycle progression rather than the transcription cycle^21–25^.

Our analysis of genetic dependency data also suggested there may be certain contexts in which other members of the tCDK family would have relevance for cancer therapy. For example, we found that CDK12 is essential in ~ 30% of cancer cell lines with average effects that were similar to those of CDK4 and CDK6. Previous studies showed genetic suppression of CDK12 activity is anti-proliferative in colorectal cancer^65^ and hepatocellular carcinoma^37^ cells. Although our results showed CDK12 essentiality is not dependent on cancer lineage, further work is needed to determine genetic and/or molecular features associated with sensitivity to loss of CDK12 activity. Interestingly, treatment with THZ531, an inhibitor of both CDK12 and CDK13, has been shown to elicit a synthetic lethal response in Ewing Sarcoma cells expressing the EWS/FLI oncogene^36^, lending credence to the potential for certain genetic aberrations to dictate CDK12 essentiality. However, throughout this study we showed differences between CDK12 and CDK13 in terms of their effects on cancer cell fitness and their genetic co-dependencies. These observations were striking given that CDK12 and CDK13 share 90.5% identity in their kinase domains^66^. The functional specialization of the paralog pairs revealed here provides important insights for drug development and indicates efforts should be also focused on disrupting interactions through domains responsible for protein–protein interactions, rather than kinase activity.

Notably, we reported several co-dependency relationships between tCDKs and unexpected cyclins. For example, the cyclins with the strongest co-dependency against CDK12 and CDK13 are cyclins H and C, respectively. Similarly, for CDK19, several alternative cyclins ranked higher than cyclin C, including cyclin L2, Y, A2, O and YL1. Although it requires appropriate experimental validation, it is possible these unexpected genetic co-dependencies are the result of novel physical interactions between these tCDKs and cyclins. For example, these relationships may represent alternative tCDK/cyclin partnerships or previously un-reported interactions of CDK/cyclin-containing complexes. Future investigations should seek to probe these potential novel relationships.

Despite the interesting results presented in this analysis, there are some potential limitations that should be considered. Foremost, any conclusions about physical interactions would need to be confirmed by biochemical assays, as it is entirely probable that two genes could have positive genetic co-dependency without being the result of direct physical interaction. We also note the overall low magnitude of some of the gene effect correlations described. However, it is important to consider the presented findings in the context of the correlation values reported for well-established functional interactions. For example, CDK8 and cyclin C are well-established biochemical partners, but their genetic co-dependency only reaches a rho of 0.40, which in turn is the strongest for CDK8 across the genome. Similarly, CDK7 and cyclin H show a rho of 0.16. Therefore, whereas these values may not be considered strong associations by conventional standards, they can be considered values representative of bona fide physical interactions. There are several potential reasons for these low rho values. One explanation is that these measurements were performed across 1070 diverse cancer cell types, which due to the heterogeneity of cancer, likely have subpopulations with genetic and/or molecular perturbations that confound these correlation values. There also appears to be a limit of detection for extreme negative gene effect scores, such that genes with strong negative gene effects may have weaker correlations with most others due to a limited dynamic range of gene effect scores. This is demonstrated by *CDK7*, which has a distribution of gene effect scores that cluster tightly around -2. Another driver of these low rho values may be that in some instances a biochemical partnership role can be filled by multiple factors. For example, in the case of CDK9 which can interact with both cyclins T1 and T2. Indeed, it is the case that upon knockout of one of the T cyclins, the other could interact with CDK9 in a compensatory role which could weaken both sets of gene effect correlations. Hence, it could be expected that knocking out both T cyclins would give a more similar effect to CDK9 depletion. The opposite scenario, wherein one cyclin has multiple tCDK targets, is also potentially true. This may be the case for cyclin C, which interacts with both CDK8 and CDK19. Finally, another type of relationship that may be missed in this analysis are associations between genes that could have opposite interactions, depending on context. For example, in the case of CDK8, the positive and negative effects on transcriptional processes may cancel out any apparent co-dependencies with Mediator genes.

There were several instances where we reported relationships between tCDKs that showed essentiality in at least some proportion of cell lines versus genes that did not produce many essential gene effect scores (less than − 0.5). For example, this was the case with CDK12, which is essential in ~ 30% of cancer cells, and AFF3 which is essential in just a few cells. It is important to note that the value of − 0.5 for essentiality is an arbitrary cutoff set by the DepMap consortium. This does not mean genes that produce negative gene effect scores do not have any net influence on cell proliferation or survival. For example, *AFF3* knockout produces negative gene effect scores in myriad cell lines. Although few are less than − 0.5, this could be interpreted that AFF3 has an influence on cell proliferation and survival, but not an essential influence that causes cell death upon its knockout. The potential for a relationship between CDK12 and AFF3, for example, is strengthened by the fact that the correlation of gene effect holds true for both negative and positive gene effect scores. Together, these types of observations could be interpreted that in some proportion of cancer cell lines, CDK12 and AFF3 may have synergistic effects on cell proliferation and survival, potentially affording synthetic lethal opportunities. Although this would need to be rigorously examined by appropriate experimentation.

It is also important to acknowledge there is a lack of details for some of the drugs analyzed in the GDSC analysis. For example, the compound THZ-2-102-1, reported as a CDK7 inhibitor, was derived from a similar molecule (THZ1), which also inhibits CDK12 and CDK13. To our knowledge, it is unknown if THZ-2-102-1 shares this specificity profile. Thus, there is potential that our analysis of CDK7 pharmacological inhibition may be confounded by off-target effects on CDK12 and/or CDK13. Moreover, despite the promise shown by the CDK9 inhibitors, CDK9_5038 and CDK9_5576, we were unable to find additional studies on these compounds, particularly those investigating their target specificity.

Overall, our cross-omics analysis of genetic dependency data from over 1000 cancer cell lines, tumor mRNA expression and prognosis data from the TCGA project, and pharmacologic sensitivity data from more than 800 cancer cell lines indicates CDK7 and CDK9 are putative oncogenic tCDKs acting through diverse mechanisms. In contrast, CDK8, CDK10, CDK12 and CDK13 are conditionally oncogenic and future investigations are warranted to determine cellular perturbations conferring their oncogenicity. These results provide a resource for myriad follow up investigations including those seeking to further develop tCDKs as targets in cancer therapy.

## Methods

### Multiple sequence alignment

Peptide sequences were downloaded from https://www.uniprot.org on 03/25/22. Multiple sequence alignment and the resulting phylogenetic analysis was performed using Clustal Omega from the European Molecular Biology Laboratory’s (EMBL) European Bioinformatics Institute (EBI) web portal (https://www.ebi.ac.uk/Tools/msa/). Visualizations of phylogenetic trees were downloaded directly from the EBI web portal.

### DepMap genetic co-dependency analysis

Chronos-corrected gene effect scores from the DepMap project (release 22Q1) were obtained from https://depmap.org/portal/download/all/ on 03/24/2022. The 22Q1 release covers gene effect scores of 17,386 genes across 1070 cell lines. These gene effect scores are calculated based on the effect size of a gene knockout normalized against the distribution of pan-essential and non-essential genes. Genes with scores less than − 0.5 are considered essential, effect scores less than -1.0 are considered a strong cytotoxic effect. Genetic co-dependency between genes was determined by computing pairwise Spearman correlations and p-values across all genes in the DepMap dataset. Benjamini–Hochberg correction was used to control for false-discovery rate (q-values). Significant (q < 0.1) positive Spearman correlations were considered co-dependencies, whereas significant negative correlations were considered inverse co-dependencies. Correlation matrices were visualized by unsupervised hierarchical clustering. Computations were performed in R (R v4.0.3/RStudio 1.4.1103), rankings and visualizations were made using the tidyverse (v1.3.0), ggplot2 (v3.3.3) and ComplexHeatmap (v2.6.2) packages.

### GDSC inhibitor sensitivity analysis

Area under the curve (AUC) data from fitted growth-response models from the GDSC project (released on 02/25/20) were obtained from https://www.cancerrxgene.org/downloads/bulk_download on 07/29/21. Drug and cell line combinations with duplicate entries that had an AUC range of > 0.2 were removed from the analysis and mean AUC was used for subsequent analysis. Drugs with data from < 70% of cell lines in the datasets were excluded. Transcripts per million (TPM) RNAseq gene expression data for cell lines in GDSC were obtained from https://depmap.org/portal/download/all/ (release 21Q2, on 08/17/21). Pairwise Spearman correlations and p-values were computed across all drugs in the GDSC datasets, and for GDSC drugs against matched RNA-expression data. Benjamini–Hochberg correction was used to control for false-discovery rate. Genes with expression negatively correlated with drug treatment were considered sensitivity genes, whereas those that positively were considered resistance genes. Correlation matrices were visualized as heatmaps along with unsupervised hierarchical clustering. Computations were performed in R (R v4.0.3/RStudio 1.4.1103), rankings and visualizations were made using the tidyverse (v1.3.0), ggplot2 (v3.3.3) and ComplexHeatmap (v2.6.2) packages.

### TCGA analysis

Clinical outcome data for TCGA patients were sourced from Liu et al.^42^ and normalized RSEM RNA-seq expression data were downloaded from the Broad GDAC (https://gdac.broadinstitute.org/) on 10/11/19 using the firehose_get tool. Iterative Kaplan–Meier log-rank testing was performed as previously described^67^ to determine optimal stratification of tumor samples into high- and low- expression strata. Starting at the 10^th^ and 90^th^ percentiles, and progressing in single-percentile iterations, we performed log-rank testing for differences in progression-free interval using the survminer (v0.4.6), survival (v2.44-1.1), and purrr (0.3.3) R packages. Tests without unique sample partitions and less than 10 events in either group (high or low) were excluded. To control for false-discovery rate (FDR), Benjamini–Hochberg correction was used to adjust p-values. Within each cancer type, the stratification with the lowest p-value was selected and Kaplan–Meier survival plots were created using the survminer (v0.4.6) package.

### Gene set enrichment analysis

Pre-ranked Gene Set Enrichment Analysis (GSEA)^68^ with Hallmark gene sets^69^ (obtained from Molecular Signatures Database, http://www.gsea-msigdb.org/gsea/index.jsp) was performed using Spearman correlations as the ranking metric. Computations were carried out in R using the fgsea package (v1.16.0). To account for large differences in rho values across kinases, we multiplied the normalized enrichment scores by the maximum absolute gene effect rho value for each kinase. These analyses were carried out for tCDK gene effect correlations and correlations of GDSC drug effects versus matched RNA expression. Heatmaps were generated using the ComplexHeatmaps package (v2.6.2).

## Supplementary Information

Supplementary Information 1.

Supplementary Information 2.

Supplementary Information 3.

Supplementary Information 4.

Supplementary Information 5.

Supplementary Information 6.

Supplementary Information 7.

Supplementary Information 8.

## Data Availability

All data generated or analyzed during this study are included in this published article (and its supplementary information files). Additionally, links to publicly available datasets used are provided. Peptide sequences were downloaded from https://www.uniprot.org. Chronos-corrected gene effect scores from the DepMap project and transcripts per million (TPM) RNAseq gene expression data for cell lines in GDSC were obtained from were obtained from https://depmap.org/portal/download/all/. Drug sensitivity data from the GDSC project were obtained from https://www.cancerrxgene.org/downloads/bulk_download. Normalized RSEM RNA-seq expression data for TCGA samples were obtained from https://gdac.broadinstitute.org.

## References

[1] Bradner JE, Hnisz D, Young RA (2017). Transcriptional addiction in cancer. Cell.

[2] Franco HL, Kraus WL (2015). No driver behind the wheel? Targeting transcription in cancer. Cell.

[3] Galbraith MD, Bender H, Espinosa JM (2019). Therapeutic targeting of transcriptional cyclin-dependent kinases. Transcription.

[4] Zhang M (2021). CDK inhibitors in cancer therapy, an overview of recent development. Am. J. Cancer Res..

[5] Constantin TA, Greenland KK, Varela-Carver A, Bevan CL (2022). Transcription associated cyclin-dependent kinases as therapeutic targets for prostate cancer. Oncogene.

[6] Vervoort SJ (2022). Targeting transcription cycles in cancer. Nat. Rev. Cancer.

[7] Fisher RP (2017). CDK regulation of transcription by RNAP II: Not over 'til it's over?. Transcription.

[8] Chou J, Quigley DA, Robinson TM, Feng FY, Ashworth A (2020). Transcription-associated cyclin-dependent kinases as targets and biomarkers for cancer therapy. Cancer Discov..

[9] Phatnani HP, Greenleaf AL (2006). Phosphorylation and functions of the RNA polymerase II CTD. Genes Dev..

[10] Buratowski S (2009). Progression through the RNA polymerase II CTD cycle. Mol. Cell.

[11] Zaborowska J, Egloff S, Murphy S (2016). The pol II CTD: New twists in the tail. Nat. Struct. Mol. Biol..

[12] Fant CB, Taatjes DJ (2019). Regulatory functions of the Mediator kinases CDK8 and CDK19. Transcription.

[13] Larochelle S (2012). Cyclin-dependent kinase control of the initiation-to-elongation switch of RNA polymerase II. Nat. Struct. Mol. Biol..

[14] Rimel JK (2020). Selective inhibition of CDK7 reveals high-confidence targets and new models for TFIIH function in transcription. Genes Dev..

[15] Sava GP, Fan H, Coombes RC, Buluwela L, Ali S (2020). CDK7 inhibitors as anticancer drugs. Cancer Metastasis Rev..

[16] Galbraith MD, Donner AJ, Espinosa JM (2010). CDK8: A positive regulator of transcription. Transcription.

[17] Pelish HE (2015). Mediator kinase inhibition further activates super-enhancer-associated genes in AML. Nature.

[18] Donner AJ, Ebmeier CC, Taatjes DJ, Espinosa JM (2010). CDK8 is a positive regulator of transcriptional elongation within the serum response network. Nat. Struct. Mol. Biol..

[19] McDermott MS (2017). Inhibition of CDK8 mediator kinase suppresses estrogen dependent transcription and the growth of estrogen receptor positive breast cancer. Oncotarget.

[20] Nemet J, Jelicic B, Rubelj I, Sopta M (2014). The two faces of Cdk8, a positive/negative regulator of transcription. Biochimie.

[21] Larochelle S, Pandur J, Fisher RP, Salz HK, Suter B (1998). Cdk7 is essential for mitosis and for in vivo Cdk-activating kinase activity. Genes Dev.

[22] Fisher RP (2005). Secrets of a double agent: CDK7 in cell-cycle control and transcription. J. Cell Sci..

[23] Larochelle S (2006). Dichotomous but stringent substrate selection by the dual-function Cdk7 complex revealed by chemical genetics. Nat. Struct. Mol. Biol..

[24] Larochelle S (2007). Requirements for Cdk7 in the assembly of Cdk1/cyclin B and activation of Cdk2 revealed by chemical genetics in human cells. Mol. Cell.

[25] Schachter MM (2013). A Cdk7-Cdk4 T-loop phosphorylation cascade promotes G1 progression. Mol. Cell.

[26] Veo B (2021). Transcriptional control of DNA repair networks by CDK7 regulates sensitivity to radiation in MYC-driven medulloblastoma. Cell Rep..

[27] Sánchez-Martínez C, Lallena MJ, Sanfeliciano SG, de Dios A (2019). Cyclin dependent kinase (CDK) inhibitors as anticancer drugs: Recent advances (2015–2019). Bioorg. & Med. Chem. Lett..

[28] Roskoski R (2019). Cyclin-dependent protein serine/threonine kinase inhibitors as anticancer drugs. Pharmacol. Res..

[29] Marineau JJ (2022). Discovery of SY-5609: A selective, noncovalent inhibitor of CDK7. J. Med. Chem..

[30] Christensen CL (2014). Targeting transcriptional addictions in small cell lung cancer with a covalent CDK7 inhibitor. Cancer Cell.

[31] Zhang Y (2019). The covalent CDK7 inhibitor THZ1 potently induces apoptosis in multiple myeloma cells in vitro and in vivo. Clin. Cancer Res. Off. J. Am. Assoc. Cancer Res..

[32] Huang JR (2018). Cyclin-dependent kinase 7 inhibitor THZ2 inhibits the growth of human gastric cancer in vitro and in vivo. Am. J. Transl. Res..

[33] Zhang J (2020). Targeting super-enhancer-associated oncogenes in osteosarcoma with THZ2, a covalent CDK7 inhibitor. Clin. Cancer Res. Off. J. Am. Assoc. Cancer Res..

[34] Cidado J (2020). AZD4573 is a highly selective CDK9 inhibitor that suppresses MCL-1 and induces apoptosis in hematologic cancer cells. Clin. Cancer Res. Off. J. Am. Assoc. Cancer Res..

[35] Geng M (2019). Targeting CDK12-mediated transcription regulation in anaplastic thyroid carcinoma. Biochem. Biophys. Res. Commun..

[36] Iniguez AB (2018). EWS/FLI confers tumor cell synthetic lethality to CDK12 inhibition in Ewing sarcoma. Cancer Cell.

[37] Wang C (2020). CDK12 inhibition mediates DNA damage and is synergistic with sorafenib treatment in hepatocellular carcinoma. Gut.

[38] Nitulescu II (2017). Mediator kinase phosphorylation of STAT1 S727 promotes growth of neoplasms with JAK-STAT activation. EBioMedicine.

[39] Malumbres M, Barbacid M (2005). Mammalian cyclin-dependent kinases. Trends Biochem. Sci..

[40] Cao L (2014). Phylogenetic analysis of CDK and cyclin proteins in premetazoan lineages. BMC Evol. Biol..

[41] Dempster JM (2021). Chronos: a cell population dynamics model of CRISPR experiments that improves inference of gene fitness effects. Genome Biol..

[42] Liu J (2018). An integrated TCGA pan-cancer clinical data resource to drive high-quality survival outcome analytics. Cell.

[43] Shimada K, Bachman JA, Muhlich JL, Mitchison TJ (2021). shinyDepMap, a tool to identify targetable cancer genes and their functional connections from Cancer Dependency Map data. Elife.

[44] Jeronimo C (2016). Tail and kinase modules differently regulate core mediator recruitment and function in vivo. Mol. Cell.

[45] Warfield L, Donczew R, Mahendrawada L, Hahn S (2022). Yeast Mediator facilitates transcription initiation at most promoters via a Tail-independent mechanism. Mol. Cell.

[46] Knoll ER, Zhu ZI, Sarkar D, Landsman D, Morse RH (2018). Role of the pre-initiation complex in Mediator recruitment and dynamics. Elife.

[47] Peissert S, Schlosser A, Kendel R, Kuper J, Kisker C (2020). Structural basis for CDK7 activation by MAT1 and Cyclin H. Proc. Natl. Acad. Sci. U. S. A..

[48] Glover-Cutter K (2009). TFIIH-associated Cdk7 kinase functions in phosphorylation of C-terminal domain Ser7 residues, promoter-proximal pausing, and termination by RNA polymerase II. Mol. Cell. Biol..

[49] Luo Z (2012). The super elongation complex family of RNA polymerase II elongation factors: Gene target specificity and transcriptional output. Mol. Cell. Biol..

[50] Bacon CW, D'Orso I (2019). CDK9: A signaling hub for transcriptional control. Transcription.

[51] Decker TM (2019). Analog-sensitive cell line identifies cellular substrates of CDK9. Oncotarget.

[52] Coutton C (2019). Bi-allelic mutations in ARMC2 lead to severe astheno-teratozoospermia due to sperm flagellum malformations in humans and mice. Am. J. Hum. Genet..

[53] Tsutsui T, Fukasawa R, Tanaka A, Hirose Y, Ohkuma Y (2011). Identification of target genes for the CDK subunits of the Mediator complex. Genes Cells.

[54] Zimmerman WC, Erikson RL (2007). Polo-like kinase 3 is required for entry into S phase. Proc. Natl. Acad. Sci. U.S.A..

[55] Burrows F, Zhang H, Kamal A (2004). Hsp90 activation and cell cycle regulation. Cell Cycle (Georgetown, Tex.).

[56] Telles E, Seto E (2012). Modulation of cell cycle regulators by HDACs. Front. Biosci. (Schol Ed.).

[57] Wee P, Wang Z (2017). Epidermal growth factor receptor cell proliferation signaling pathways. Cancers.

[58] Povedano JM (2022). TK216 targets microtubules in Ewing sarcoma cells. Cell. Chem. Biol..

[59] Niwa S (2015). Kinesin superfamily proteins and the regulation of microtubule dynamics in morphogenesis. Anat. Sci. Int..

[60] Ramírez-Cosmes A (2021). The implications of ABCC3 in cancer drug resistance: Can we use it as a therapeutic target?. Am. J. Cancer. Res..

[61] Zhang Y (2017). Positive transcription elongation factor b (P-TEFb) is a therapeutic target in human multiple myeloma. Oncotarget.

[62] Ganuza M (2012). Genetic inactivation of Cdk7 leads to cell cycle arrest and induces premature aging due to adult stem cell exhaustion. EMBO J..

[63] Zhang H (2020). CDK7 inhibition potentiates genome instability triggering anti-tumor immunity in small cell lung cancer. Cancer Cell.

[64] Olson CM (2019). Development of a selective CDK7 covalent inhibitor reveals predominant cell-cycle phenotype. Cell Chem. Biol..

[65] Dieter SM (2021). Degradation of CCNK/CDK12 is a druggable vulnerability of colorectal cancer. Cell Rep..

[66] Lv L (2020). Discovery of a molecular glue promoting CDK12-DDB1 interaction to trigger cyclin K degradation. Elife.

[67] Andrysik Z, Bender H, Galbraith MD, Espinosa JM (2021). Multi-omics analysis reveals contextual tumor suppressive and oncogenic gene modules within the acute hypoxic response. Nat. Commun..

[68] Subramanian A (2005). Gene set enrichment analysis: A knowledge-based approach for interpreting genome-wide expression profiles. Proc. Natl. Acad. Sci. U. S. A..

[69] Liberzon A (2015). The Molecular Signatures Database (MSigDB) hallmark gene set collection. Cell Syst..

