# Supplementary material for: Multi-omics investigation reveals functional specialization of transcriptional cyclin dependent kinases in cancer biology
Source: Sci Rep. 2022 Dec 28;12:22505. doi: 10.1038/s41598-022-26860-1 (PMC9797569; doi:10.1038/s41598-022-26860-1)
Supplement: Supplementary file 1 — Supplementary Information 1. [file 41598_2022_26860_MOESM1_ESM.pdf]

Multi-omics investigation reveals functional specialization of transcriptional cyclin dependent kinases in cancer biology

Micah G. Donovan, Matthew D. Galbraith, and Joaquin M. Espinosa

Supplementary Information.

Supplementary Figures and Supplementary Data Files.

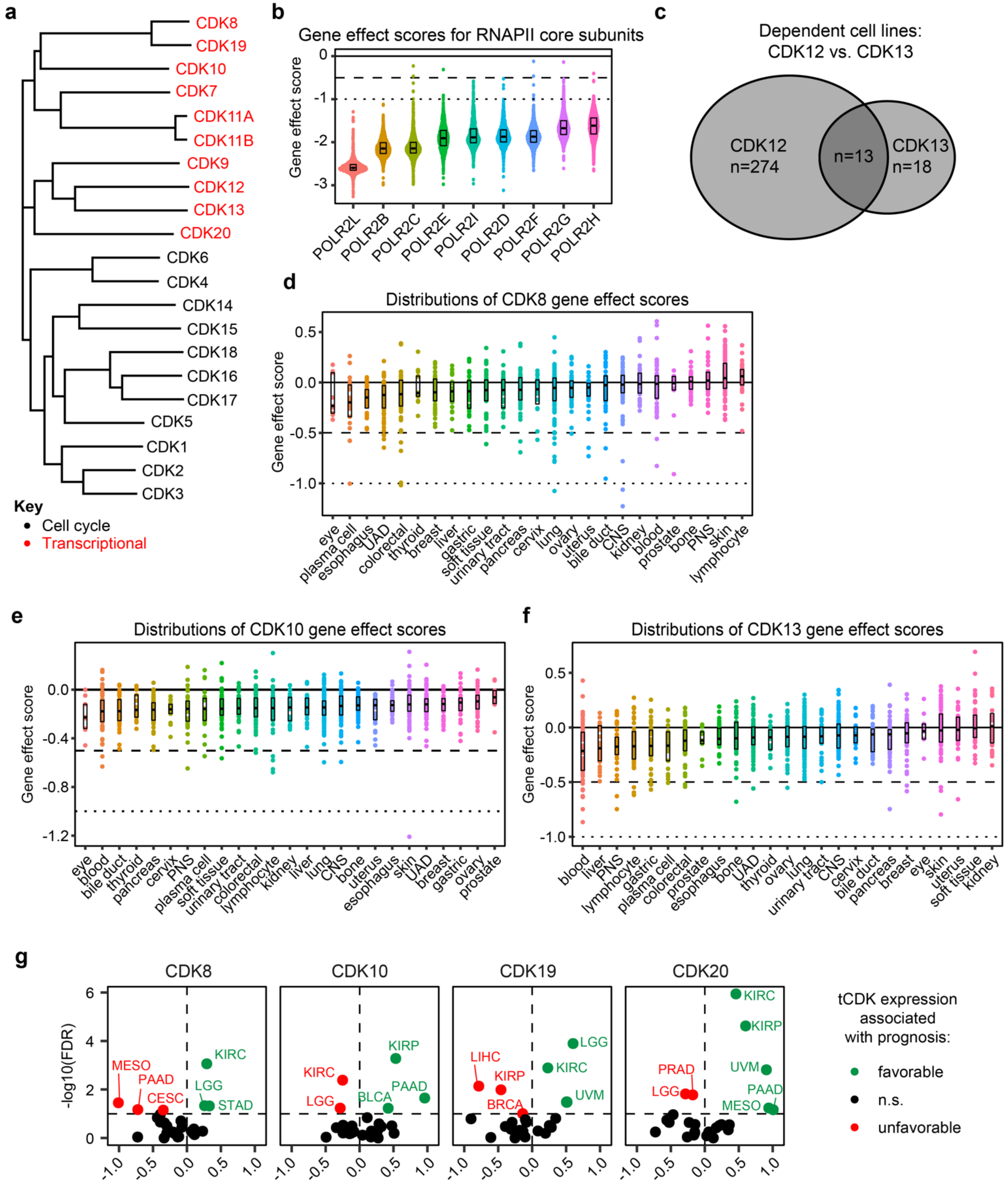

Supplementary Figure 1: Human tCDKs have diverse effects on cancer cell viability.

a. Full peptide phylogenetic sequence analysis of CDKs. Red indicates tCDKs, black indicates cell cycle-related CDKs.

b. Distributions of gene effect scores for all RNAPII subunits (*POLR2*) tested in DepMap data set. Dashed line indicates threshold for essential gene effect scores (-0.5). Dotted line indicates strong killing effect (-1.0). Box plots show median with first and third quartiles at hinges. Whiskers extend to largest/smallest values no further than 1.5*IQR from hinge.

c. Venn diagram showing cell lines in which *CDK12* and *CDK13* are essential. Overlap shows cell lines where both *CDK12* and *CDK13* are essential.

d-f. Distributions of gene effect scores for *CDK8* (d), *CDK10* (e) and *CDK13* (f) across all lineages with at least 5 representative cell lines. Box plots show median with first and third quartiles at hinges. Whiskers extend to largest/smallest values no further than 1.5*IQR from hinge.

g. Volcano plots for prognosis associated with tCDK expression in the TCGA dataset. Results show adjusted (log2) of progression-free survival (PFS) ratio vs. adjusted (-log10) false discovery rate (FDR).

a

Kinase	Reported cyclin interactions
CDK1	CCNA1/2, CCNB1/2/3
CDK2	CCNA1/2, CCNE1/2
CDK3	CCNC
CDK4	CCND1/2/3
CDK5	CCND2/3, CABLES1, CABLES2, CCNG1, CCNI, CCNI2
CDK6	CCND1/2/3
CDK7	CCNH
CDK8	CCNC
CDK9	CCNK, CCNT1/2
CDK10	CCNQ
CDK11A	CCNL1/2
CDK11B	CCNL1/2
CDK12	CCNK
CDK13	CCNK
CDK14	CCNY
CDK15	
CDK16	CCNI, CCNY, CCNYL1
CDK17	
CDK18	
CDK19	CCNC
CDK20	

b

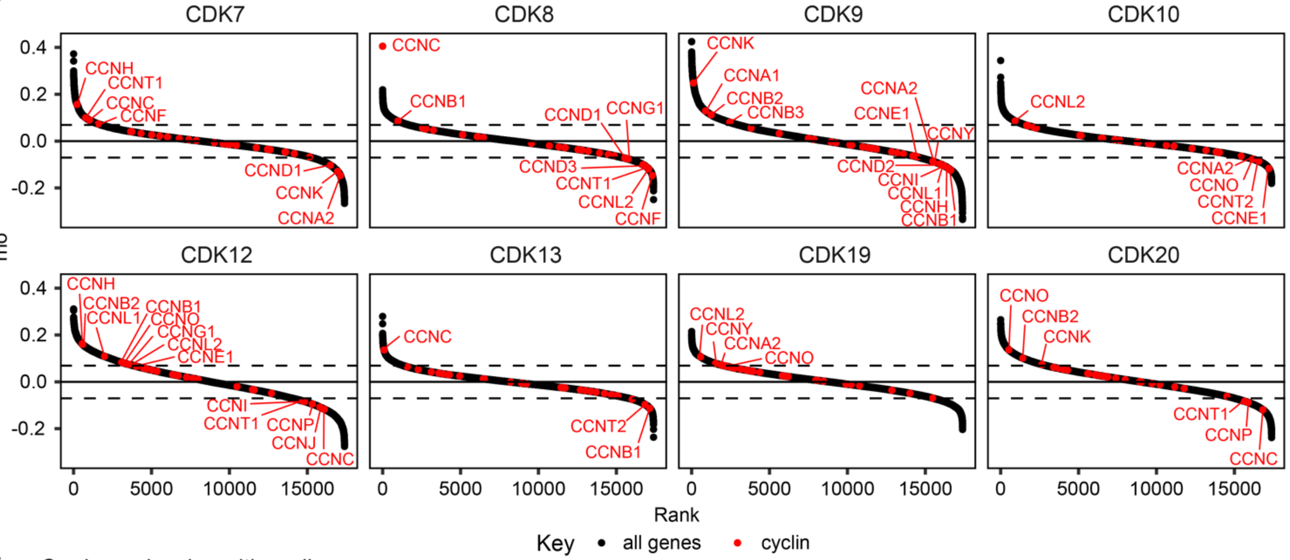

c

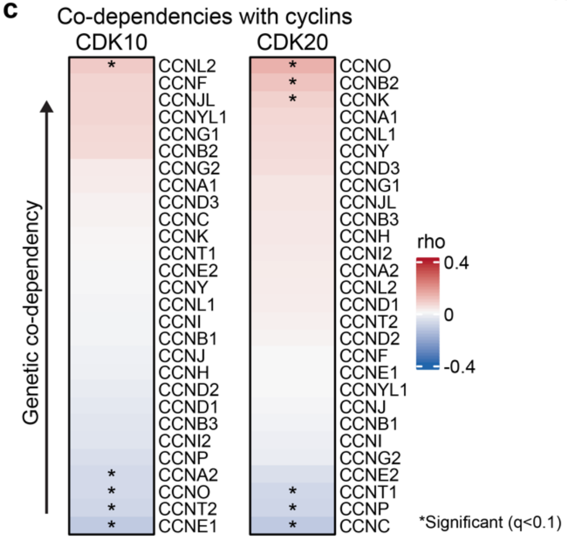

Supplementary Figure 2: Analysis of genetic co-dependencies reveals predicted versus unexpected relationships between tCDKs and their cyclins.

a. Previously reported biochemical interactions between CDKs and cyclins.

b. Ranked correlations of gene effect scores for tCDKs. Location of all cyclins depicted in red. Above and below the dashed lines denote significant (FDR<0.1) positive and negative correlations, respectively. Cyclins with significant (FDR <0.1) correlations are annotated by name.

c. Ranked gene-effect correlations between *CDK10* and *CDK20* vs. all cyclin genes in the DepMap data set. Asterisks denote significant (FDR<0.1) interactions. Arrow shows higher correlation values equate to higher co-dependency scores.

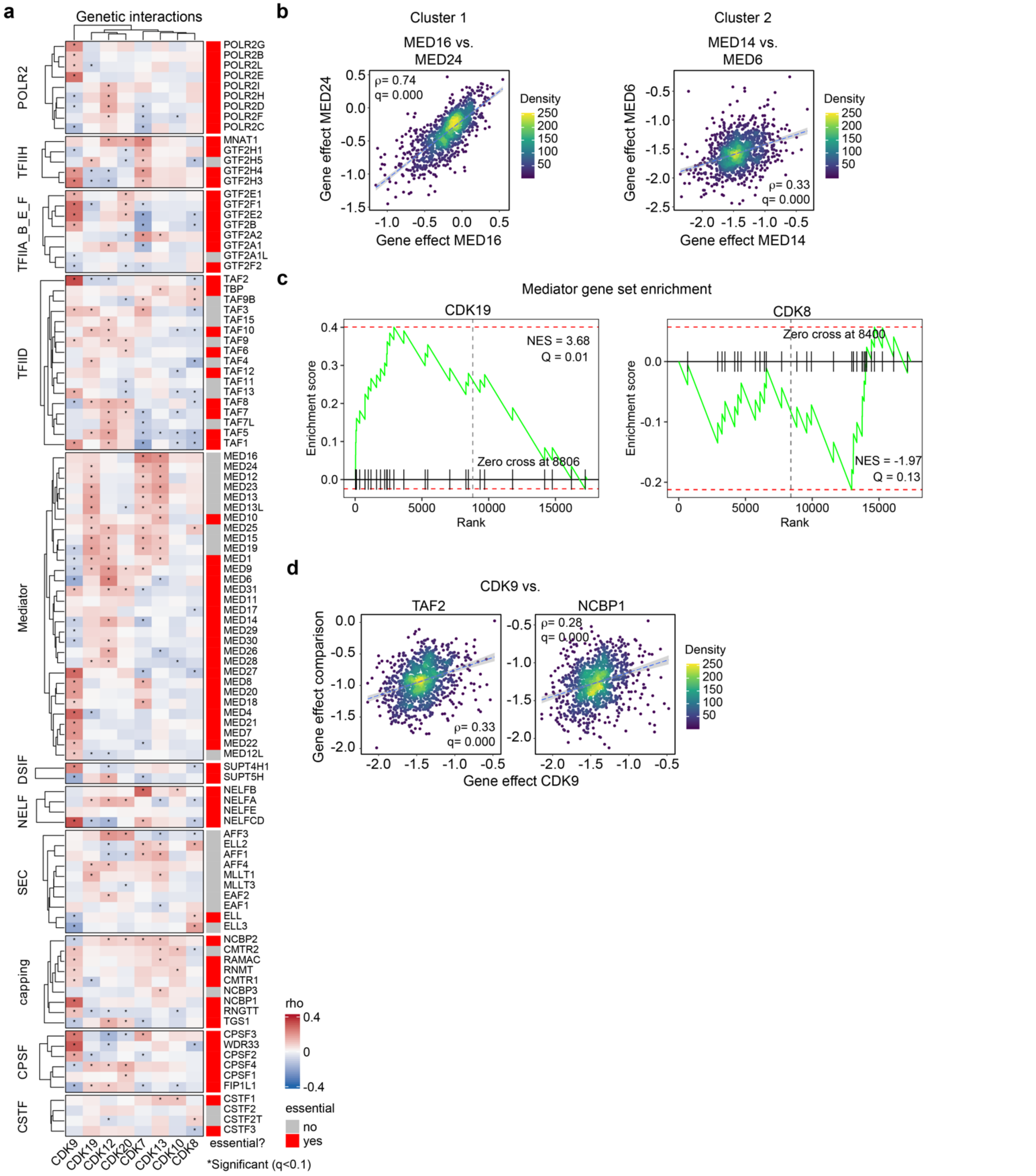

Supplementary Figure 3: tCDKs exhibit differential genetic co-dependencies with the RNAPII machinery.

a. Heatmap showing results from unsupervised clustering of tCDK gene effect correlations with genes encoding subunits of various complexes involved in RNAPII-dependent transcription. Asterisks denote significant (FDR <0.1). Essential genes (red, right-side annotation) are determined by median gene effect scores of <-0.5 across all cell lines. Dendrograms represent results from unsupervised clustering analysis.

b. Scatter plots comparing gene effect scores of *MED16* vs. *MED24* and *MED14* vs. *MED6* across all 1070 cell lines in the DepMap data set.

c. Gene set enrichment analysis of Mediator subunits for *CDK19* and *CDK8* gene effect correlations.

d. Scatter plots comparing gene effect scores of *CDK9* to *TAF2* and *NCBP1* across all 1070 cell lines in the DepMap data set.

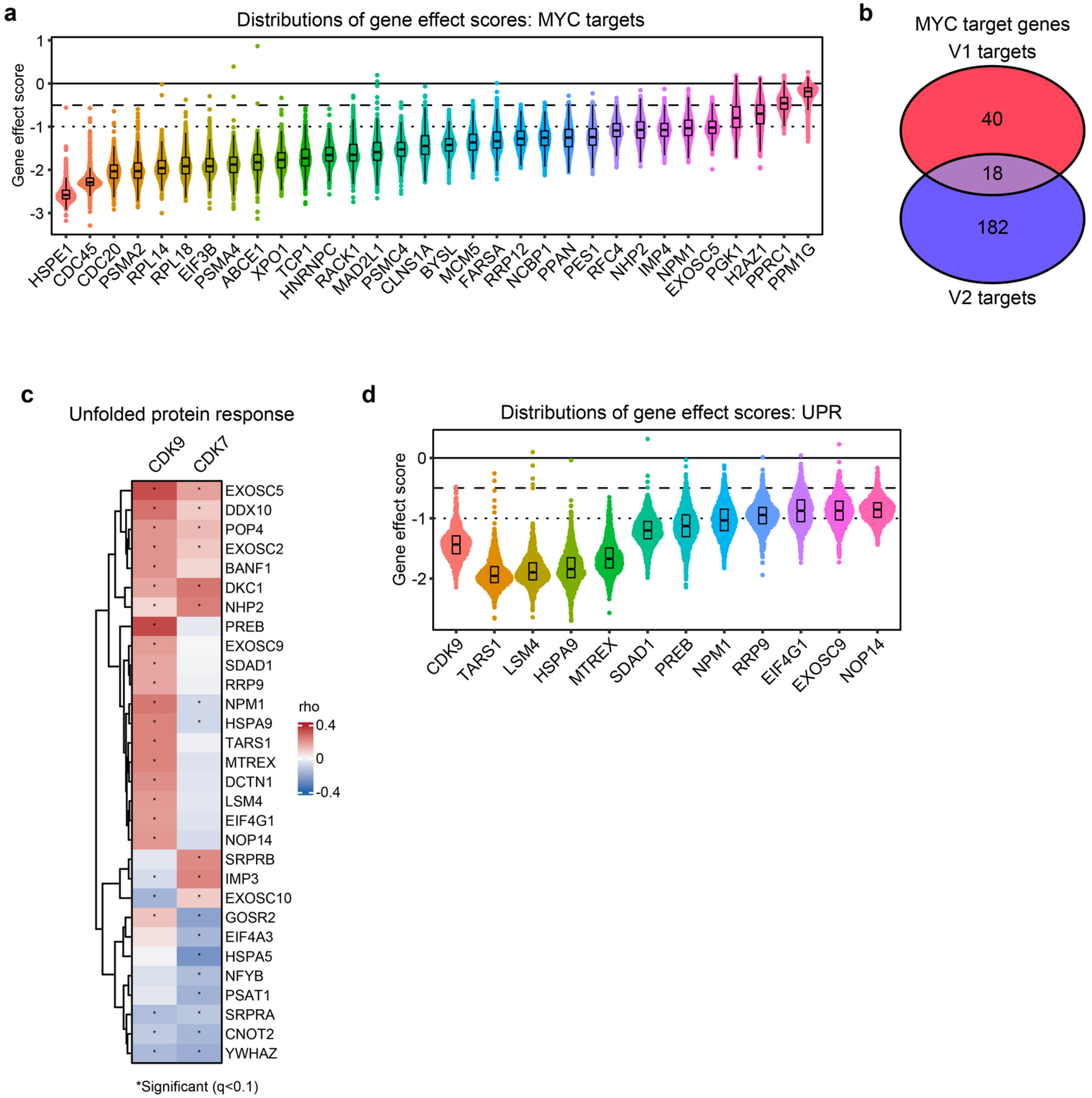

Supplementary Figure 4: Pathway analysis of genetic interactomes predicts novel functions for tCDKs.

a. Distributions of gene effect scores for select MYC targets. Dashed line indicates threshold for essential gene effect scores (-0.5). Dotted line indicates strong killing effect (-1.0). Box plots show median with first and third quartiles at hinges. Whiskers extend to largest/smallest values no further than 1.5*IQR from hinge.

b. Venn diagram showing overlap between MYC targets V1 and V2 gene sets.

c. Heatmap showing gene effect correlations for *CDK7* and *CDK9* vs. representative genes from the Hallmark Unfolded Protein Response dataset. Asterisks denote significance (FDR<0.1). Dendrograms represent results from unsupervised clustering analyses.

d. Distributions of gene effect scores for select co-dependencies of *CDK9* involved in unfolded protein response. Dashed line indicates threshold for essential gene effect scores (-0.5). Dotted line indicates strong killing effect (-1.0). Box plots show median with first and third quartiles at hinges. Whiskers extend to largest/smallest values no further than 1.5*IQR from hinge.

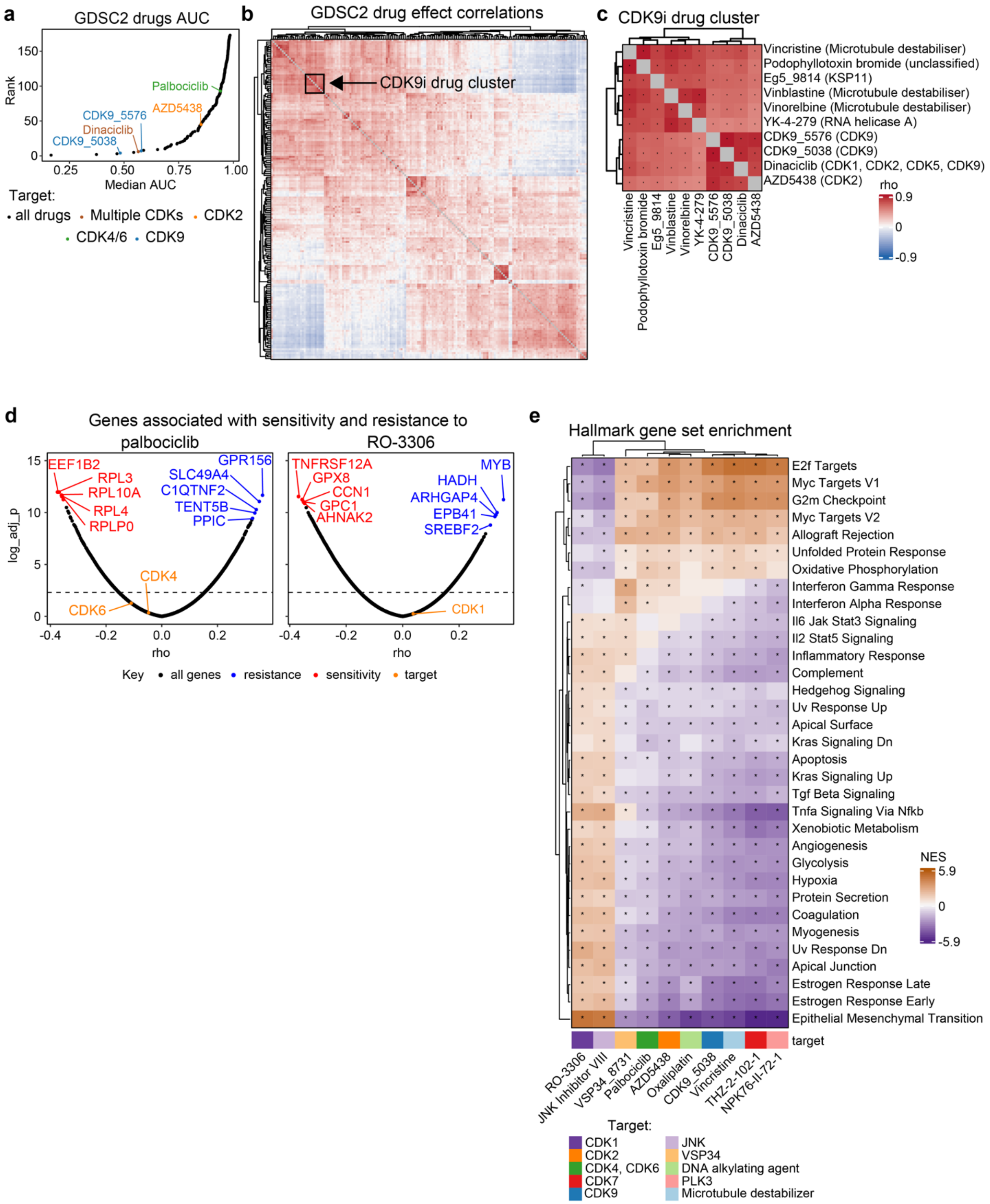

Supplementary Figure 5: Identification of gene signatures associated with sensitivity to pharmacological tCDK inhibition.

a. Ranked median area under the curve (AUC) values for all drugs in the GDSC2 data set. Drugs targeting CDKs are multi-colored and annotated.

b. Heatmap showing full matrix of correlations of AUC between all drugs in the GDSC2 dataset. Dendrograms represent results from unsupervised clustering analyses. Cluster containing the CDK9 inhibitors CDK9_5038 and CDK9_5576 is blocked off in a black square.

c. Magnification of a cluster from (b) containing the CDK9 inhibitors CDK9_5038 and CDK9_5576. Asterisks denote significance (FDR<0.1).

d. Volcano plots depicting correlations between CDK4/6 (Palbociclib) and CDK1 (RO-3306) inhibitors vs. genome-wide mRNA expression. The top 5 sensitivity (rho<0, FDR<0.1) and resistance (rho>0, FDR<0.1) genes are shown in red and blue, respectively. Drug targets are highlighted in orange.

e. Heatmap showing normalized enrichment scores (NES) from Hallmark gene set enrichment analysis of ranked drug AUC vs. gene expression correlations. Asterisks denote significant (FDR<0.1). Dendrograms represent results from unsupervised clustering analysis.

Legends for Supplementary Data files.

Supplementary Data 1.

A-S) Tabs “CDK(1-20)” - Genetic dependency data for CDKs. Each tab represents and individual CDK (1-20). Gene effect scores for all 1070 cell lines are provided with cell line name and lineage.

Supplementary Data 2.

A-H) Tabs “CDK(7-10, 12-13, 19-20)” – Association between prognosis and tCDK expression through analysis of human tumor samples from the The Cancer Genome Atlas dataset (TCGA). For each cancer type, prognosis, progression-free survival ratio (PFS ratio) and adjusted p value (padj, i.e., q value) is provided.

Supplementary Data 3.

A-S) Tabs “CDK(1-20)” - Ranked gene effect correlations for CDKs (1-20). Spearman rho values are presented with their p values (pval) and adjusted p values (adjusted pval, i.e., q value). Both positive and negative rank for each gene is also provided.

Supplementary Data 4.

A) Tab “Spearman rho values” - Spearman correlation matrix for gene effect scores of tCDKs and RNAPII-related genes.

B) Tab “Adjusted Spearman p values” - Adjusted p values matrix for gene effect scores of tCDKs and RNAPII-related genes.

Supplementary Data 5.

A-H) Tabs “CDK(7-10, 12-13, 19-20)” - Hallmark gene set enrichment analysis (GSEA) of tCDK gene effect correlations. Each tab represents and individual CDK. For each pathway, enrichment score (ES), normalized enrichment score (NES), p value (pval), adjusted p value (padj, i.e., q values), and adjusted

NES (NES adj) are provided. NES adj was calculated by NES * absolute value of the maximum gene effect correlation (|max rho|) for that tCDK.

Supplementary Data 6.

A-M) Tabs "[drug name]" - Spearman correlations for drug effects. Correlations were completed using area under the curve (AUC) values. Each tab represents a different drug of interest. Spearman rho values are presented with their p values (pval) and adjusted p values (padj, i.e., q value). Putative targets, as reported by GDSC, are provided.

Supplementary Data 7.

A-J) Tabs "[drug name]" - Spearman correlations for drug effect vs. mRNA expression. Each tab represents a different drug of interest. Spearman rho values are presented with their p values (pval) and adjusted p-values (i.e., q values).
